# A remarkable effect of the combination of probabilistic peer-punishment and coevolutionary mechanism on the evolution of cooperation

**DOI:** 10.1038/s41598-017-12742-4

**Published:** 2017-09-29

**Authors:** Tetsushi Ohdaira

**Affiliations:** Institute of Information and Media, Aoyama Gakuin University, 5-10-1 Fuchinobe, Chuo-ku, Sagamihara-city, Kanagawa 252-5258 Japan

## Abstract

In the previous studies, the author has proposed the probabilistic peer-punishment based on the difference of payoff, and presented that the proposed peer-punishment utilizes its mechanism for preventing antisocial punishment like retaliation of a defector on a cooperator, effectively enhances the evolution of cooperation, and greatly increases the average payoff of all players in various parameters regarding static three types of topology of connections. Here, this study introduces both activities of breaking and creating connections of every player based on his/her preference to the model of the proposed peer-punishment. Every player will keep connections with his/her preferable players, whereas he/she will frequently break connections with his/her dissatisfied other players. Therefore, the new model of this study is the combination of probabilistic peer-punishment and coevolutionary mechanism that not only strategy of players but also connections between players evolve. This study discovers new knowledge that such combination induces high-level evolution of cooperation and great increase of the average payoff of all players in the condition where cooperation is hard to evolve.

## Introduction

The author has proposed the probabilistic peer-punishment based on the difference of payoff in the previous study^1^, and revealed that when strategy of players evolves, the proposed peer-punishment effectively enhances the evolution of cooperation, and greatly increases the average payoff of all players in the limited condition. Further investigation^2^ shows that the proposed peer-punishment utilizes its mechanism for preventing antisocial punishment like retaliation of a defector on a cooperator, effectively enhances the evolution of cooperation, and greatly increases the average payoff of all players in various parameters regarding static three types of topology of connections that do not change during each simulation run. Players should make the difference of payoff small for the evolution of cooperation by utilizing the proposed peer-punishment because those previous studies^1,2^ employ cumulative payoff from all matches. Chen and Perc^3^ show similar knowledge that absolute (cumulative) payoff requires high-degree players to be punished stronger than low-degree players. Note that degree means the number of connections of every player. Szabó and Szolnoki^4^ also present similar result that players achieve the highest total payoff when sharing their payoff fraternally in the spatial evolutionary game with the myopic strategy update rule.

The main trait of the proposed peer-punishment is that a player determines whether he/she punishes another player or not according to the dynamically changing probability based on the difference of payoff between them. The probability of punishment of some previous studies^5–7^ is fixed among players, and does not change depending on the difference of payoff between punishing and punished players. Szolnoki *et al*.^8,9^ propose the notion of emotional profiles that seems to be conceptually like the proposed peer-punishment. However, such profiles consist of only two types of emotion. They introduce sympathy and envy as the two emotional profiles that determine the strategy of every player, and define such profiles as the probability that every player cooperates with players having lower and higher payoff, respectively^8^. They also consider the imitation of emotional profiles of neighbor players instead of pure strategy^9^. Szolnoki and Perc^10^ consider the conditional punishment that is not based on the difference of payoff between punishing and punished players, but proportional to the number of other conditional and unconditional punishers within the group. Regarding other cases utilizing probability, Chen *et al*.^11^ show that the introduction of probabilistically monitoring defectors and implicated punishment is indeed effective in realizing cooperation in infinite and finite well-mixed populations. In implicated punishment, each player of a group is punished regardless of his/her strategy once a defector is detected within that group. They also present that the addition of peer-punishment further promotes the evolution of public cooperation. Perc *et al*.^12^ systematically review the main results obtained in the realm of statistical physics of human cooperation. They describe that probabilistically sharing the responsibility to sanction defectors can solve the problem of costly punishment in the section regarding adaptive punishment.

The proposed peer-punishment has a remarkable effect on the evolution of cooperation regarding static three types of topology of connections as noted before. On the other hand, some studies prove that coevolutionary mechanism of strategy of players and connections between players facilitates cooperation^13–30^. For example, either random or intentional rewiring process contributes to cooperation^13–16^. Double resonance phenomenon, i.e. slowly varying topology of connections and additive random payoff disturbances, facilitates cooperation^17^. Pacheco *et al*.^18^ discuss the difference in the capability of searching new connections of players, considering the relation between the dynamics of strategy of players and that of connections between players. Szolnoki and Perc^19^ show that another simple coevolutionary mechanism of adoption of successful strategy and instructive behavior may lead to highly heterogeneous distributions of such behavior contributing cooperation. Szolnoki *et al*.^20^ show that there is an optimal maximal degree for cooperation utilizing coevolutionary mechanism. Poncela *et al*. employ the mechanism of preferential attachment that a new player makes connections to either the randomly selected player or the player succeeded in the past^21^, and show that such mechanism generates topology of connections between players where cooperation survives^22^. Szolnoki and Perc reveal that coevolutionary mechanism of adoption of new strategy and either breaking or creating connections between players after some rounds of game has a strong effect on cooperation^23^, and supports cooperation within entire range of temptation to defect^24^. Van Segbroeck *et al*.^25,26^ newly introduce the diversity to adverse connections between players, and show how such coevolutionary mechanism is beneficial to entire population when myopic players act for their payoff. Zhang *et al*.^27^ show that the heterogeneity of topology of connections and coevolutionary mechanism attain high-level cooperation in public goods game interactions. Lee *et al*.^28^ investigate the case where parameters of payoff matrix and connections between players coevolve with game interactions. Perc and Szolnoki^29^ explain that coevolutionary mechanism influences connections between players, capability of reproduction of players, their reputation, mobility or age. Perc *et al*.^30^ review recent advances in the study of evolutionary dynamics of group interactions on structured populations including coevolutionary mechanism. They also compare these results with those obtained on well-mixed populations.

Here, this study introduces both activities of breaking and creating connections of every player based on his/her preference to the model of the proposed peer-punishment^1,2^. Every player will keep connections with his/her preferable players, whereas he/she will frequently break connections with his/her dissatisfied other players. Therefore, the new model of this study employs the proposed peer-punishment, and includes coevolutionary mechanism that not only strategy of players but also connections between players evolve. No previous studies have investigated an effect of the combination of probabilistic peer-punishment and coevolutionary mechanism on the evolution of cooperation. This study exhibits that such combination has a remarkable effect on the evolution of cooperation.

## Model

**Figure 1:**
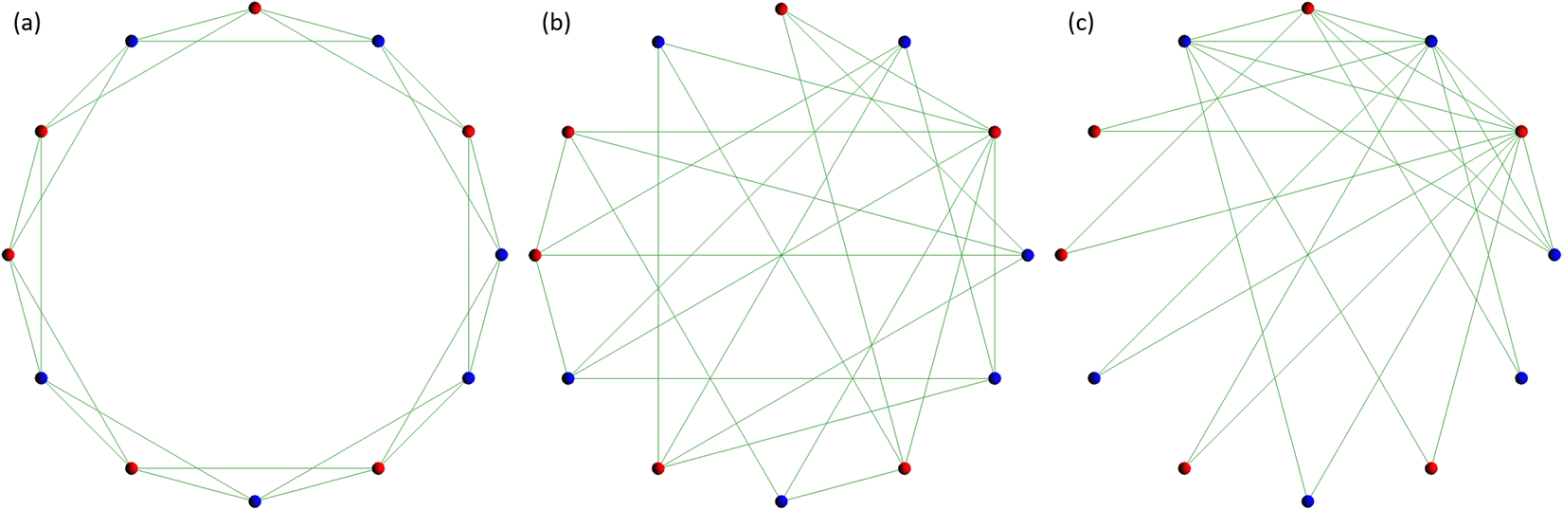
Three panels (a–c) show the samples of initial topology of connections of <*k*> = 4, i.e. (**a**) Regular, (**b**) Random, and (**c**) Scale-free. They are defined as one dimensional lattices of periodic boundary conditions, and a vertex exhibits a player. Defectors are colored red and cooperators are colored blue. Note that this figure has only 12 players for the intelligibility.

The model of this study is basically based on the proposed model of the previous studies^1,2^. As shown in Fig. 1, initial topology of connections defining the relationship of every player is following three types, i.e. (a) Regular^31^, (b) (Completely) Random^31^, and (c) Scale-free known as the Barabási-Albert model^32^. They are defined as one dimensional lattices of periodic boundary conditions, and a vertex exhibits a player. The degree (the number of connections) of player *i* is *k*(*i*). The average of *k*(*i*) (〈*k*〉) can be expressed as $< k > =\frac{1}{N}\sum _{1\le i\le N}k(i)$. The author describes the detail of the construction of each type of the topology of connections in the Methods of the previous study^1^. Figure 1 shows the samples of initial topology of connections of <*k*> = 4. Note that this figure has only 12 players for the intelligibility.

The strategy of player *i* (*s*(*i*)) is expressed as either (0 1) (defector) or (1 0) (cooperator) utilizing unit vectors. Player *i* plays the prisoner’s dilemma game with the other players connected with him/her, and then acquires the cumulative payoff *P*(*i*) from all games (matches). When *N* is the number of all players, player *j* is the opponent player of player *i* (*i *≠ *j*, 1 ≤ *i*, *j* ≤ *N*), and s(*j*) and *P*(*j*) is the strategy and payoff of player *j*, respectively, we can express *P*(*i*) as the following equation (1) utilizing the payoff matrix *A*. *O*(*i*) is the set of the other players connected with player *i*, and *b* is the temptation to defect in the prisoner’s dilemma game. The initial ratio of the number of defectors to the number of cooperators is approximately one to one, and they are randomly distributed in every simulation run following the previous studies^1,2^.

1 $$\begin{array}{c}P(i)=\sum _{j\in O(i)}s(i)As{(j)}^{T}\\ (i\ne j,1\le i,j\le N),\quad A=(\begin{array}{cc}1 & 0\\ b & 0\end{array})\quad (1 < b\le 2)\end{array}$$

Player *i* compares his/her payoff (*P*(*i*)) with the payoff of the opponent player *j* (*P*(*j*)), and he/she inflicts the cost *rP*(*i*) on the opponent player *j* with the probability *q*_*i*_(*j*) expressed as the following equations (2) and (3) when *P*(*i*)(1-*rn*(*i*)) > 0 and *P*(*i*) < *P*(*j*) ≤ 2 *P*(*i*) hold. Note that *r* is the coefficient of punishment (0 ≤ *r* ≤ 1), and *n*(*i*) is the number of players *j* $\in$ *O*(*i*) that satisfies both *P*(*j*) > *P*(*i*) and *s*(*j*) = (0 1) (defector). When *P*(*i*)(1-*rn*(*i*)) > 0 and *P*(*j*) > 2 *P*(*i*) hold, *q*_*i*_(*j*) equals 1. *P*(*i*)’ and *P*(*j*)’ cannot be negative because the decrease of payoff by punishing and punished is independently calculated regarding all players, and finally *P*(*i*)’ is set to 0 when it becomes a negative value. The proposed peer-punishment does not work in the case of *r* = 0 and 1 as described in the previous study^2^.

2 $${q}_{i}(j)=\frac{P(j)-P(i)}{P(i)},P(i) > 0$$

3 $$\begin{array}{c}P(i)\text{'}=P(i)-rP(i)\\ P(j)\text{'}=P(j)-rP(i)\end{array}$$

When all punishing and punished activities are finished, player *i* adopts the strategy of player *j*_max_ $\in$ *i* $\cup$ *O*(*i*) for his/her strategy of the matches of the next generation as the following equation (4). When two or more players have the maximum payoff, player *i* randomly adopts the strategy of one of them^1,2^. This adoption of new strategy is synchronously executed regarding all players. The definition of one generation is described in the final paragraph of this section.

4 $$\begin{array}{c}s{(i)}^{\text{'}}=s({j}_{\max })\,j{}_{\max }\in i\cup O(i)\\ P{({j}_{\max })}^{\text{'}}=\,\max ({P}^{\text{'}}\in i\cup O(i))\end{array}$$

After the adoption of new strategy regarding all players, they break existing connections and create new connections. Both activities of breaking and creating connections are basically based on the previous studies^18,25^, however, some modification is added to both activities. The detail of that modification is as follows.

Firstly, regarding the activity of breaking connections, the previous studies^18,25^ focus on adverse (unwanted) connections between players, and introduce the uniform expression of the probability *r*(*i*) that player *i* breaks his/her connection to player *j* $\in$ *O*(*i*) as $r(i)=\frac{1}{2}({\gamma }_{i}+{\gamma }_{j})$, where *γ*_*i*_ (respectively, *γ*_*j*_) is the peculiar probability at which player *i* (respectively, player *j* $\in$ *O*(*i*)) wants to break the connection with player *j* $\in$ *O*(*i*) (respectively, player *i*). On the other hand, this study, considering the pairing patterns of players *i* and *j*, precisely defines the activity of breaking connections as follows. (1) When both players *i* and *j* $\in$ *O*(*i*) are cooperators, player *i* breaks his/her connection to player *j* with the probability of minimum *γ*_*i*_ (i.e. *γ*_*S*_). (2) When player *i* is a cooperator, player *j* $\in$ *O*(*i*) is a defector, and *γ*_*i*_ is less than or equal to *γ*_*j*_ (or player *j* $\in$ *O*(*i*) is a cooperator, player *i* is a defector, and *γ*_*j*_ is less than or equal to *γ*_*i*_), player *i* breaks his/her connection to player *j* with the probability *γ*_*S*_. (3) In any case not covered by those conditions, player *i* breaks his/her connection to player *j* $\in$ *O*(*i*) with the probability $r(i)=\frac{1}{2}({\gamma }_{i}+{\gamma }_{j})$.

**Figure 2:**
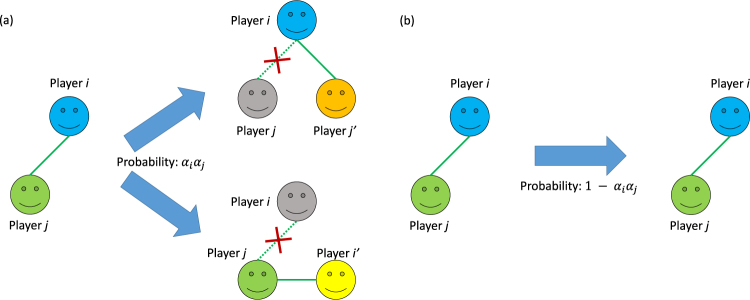
The illustration of breaking and creating connections between players *i* and *j*. When either player *i* or *j* breaks his/her connection to player *j* or *i*, on the one hand either player *i* or *j* creates his/her new connection to randomly selected another player *j*′ $\notin$ *O*(*i*) or *i*′ $\notin$ *O*(*j*) with the probability *α*_*i*_ *α*_*j*_ (see the panel (a)), but on the other hand players *i* and *j* create their connections with the probability 1-*α*_*i*_ *α*_*j*_ again (see the panel (b)).

Secondly, regarding the activity of creating connections, the previous studies^18,25^ describe that player *i* has his/her propensity *α*_*i*_ to create new connections, and creates his/her new connection to another player *j*′ $\notin$ *O*(*i*) with the probability *α*_*i*_ *α*_*j*′_. In this study, unlike the previous studies^18,25^, every player creates new connections as follows. When either player *i* or *j* $\in$ *O*(*i*) breaks his/her connection to player *j* or *i*, on the one hand either player *i* or *j* creates his/her new connection to randomly selected another player *j*′ $\notin$ *O*(*i*) or *i*′ $\notin$ *O*(*j*) with the probability *α*_*i*_ *α*_*j*_, but on the other hand players *i* and *j* create their connections with the probability 1-*α*_*i*_ *α*_*j*_ again. That activity of creating new connections considers the case where we cannot break connections to others because of the geographical or social restrictions (e.g. we cannot easily change our place of residence or jobs) although we decide to break such connections once. Unlike the previous studies^18,25^, each value of <*k*> regarding initial topology of connections is conserved in the dynamics of connections of this study. Figure 2 is the illustration of breaking and creating connections between players *i* and *j*.

The setting of parameters regarding computer simulation is as follows. Firstly, following the previous studies^1,2^, *N* = 1000, *b* = 1.5, and the initial ratio of the number of defectors to the number of cooperators approximately equals one to one. Defectors and cooperators are randomly distributed in every simulation run. Secondly, following the previous studies^18,25^, this study determines that the minimum *γ*_*i*_ (*γ*_*S*_) and the maximum *γ*_*i*_ (*γ*_*F*_) equal 0.25 and 0.75, respectively. The peculiar probability at which player *i* wants to break connections (*γ*_*i*_) randomly falls into the *M* = 50 subdivisions of the interval [0.25, 0.75] so that the number of players with the same peculiar probability of breaking connections is equal regarding all subdivisions. Regarding the propensity *α*_*i*_ to create new connections, every player has the same propensity *α* = *α*_*i*_ = 0.4 following the previous studies^18,25^. This study defines the process composed of (1) all matches of the prisoner’s dilemma game, (2) all punishing and punished activities, (3) the adoption of new strategy regarding all players, and (4) all activities of breaking and creating connections as one generation. Every simulation run lasts until the number of generations reaches 300. The following results are the average of 20 simulation runs, and have error bars (SD, standard deviation) if necessary.

## Results

**Figure 3:**
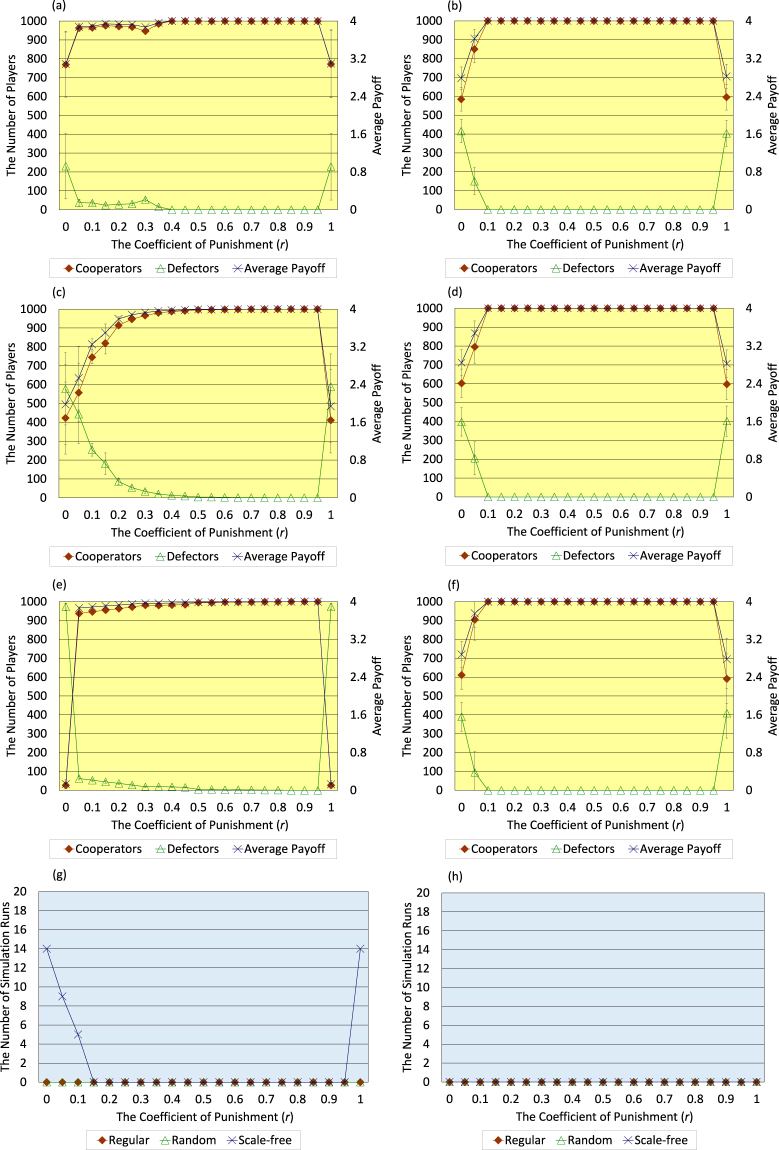
Six panels (a–f) show the dependence of the number of cooperators, the number of defectors, and the average payoff of all players in the 300 generation on the coefficient of punishment (*r*) regarding the cases where the initial topology of connections of <*k*> = 4 is regular (**a**,**b**), random (**c**,**d**), and scale-free (**e**,**f**). Bottom two panels (**g**,**h**) show the dependence of the number of simulation runs that 95 percent or more players come to defectors in the 300 generation (i.e. the number of defector-prevailing simulation runs) on the coefficient of punishment (*r*) regarding three types of initial topology of connections of <*k*> = 4, i.e. regular, random, and scale-free. Panels of left column (**a**,**c**,**e**,**g**) indicate the results without both activities of breaking and creating connections, and panels of right column (**b**,**d**,**f**,**h**) indicate the results with those activities (error bars: SD, standard deviation). Following the previous study^2^, this study considers that the evolution of cooperation emerges when the number of defector-prevailing simulation runs is 9 or less out of 20 simulation runs, otherwise defectors defeat cooperators.

Firstly, the author describes the results regarding three cases where initial topology of connections of <*k*> = 4 is regular (Fig. 3(a,b)), random (Fig. 3(c,d)), and scale-free (Fig. 3(e,f)). Those panels exhibit the following trend; in the case with both activities of breaking and creating connections, the range of *r* (the coefficient of punishment) where high-level cooperation evolves and the average payoff of all players greatly increases is wider than such range in the case without those activities. This trend is especially exhibited in the initial random case (Fig. 3(c,d)). Moreover, as shown in Fig. 3(g,h), in the case with those activities, the number of simulation runs that 95 percent or more players come to defectors in the 300 generation (i.e. the number of defector-prevailing simulation runs) falls to 0 regarding every initial topology of connections in an entire range of *r*. Following the previous study^2^, this study considers that the evolution of cooperation emerges when the number of defector-prevailing simulation runs is 9 or less out of 20 simulation runs, otherwise defectors defeat cooperators.

**Figure 4:**
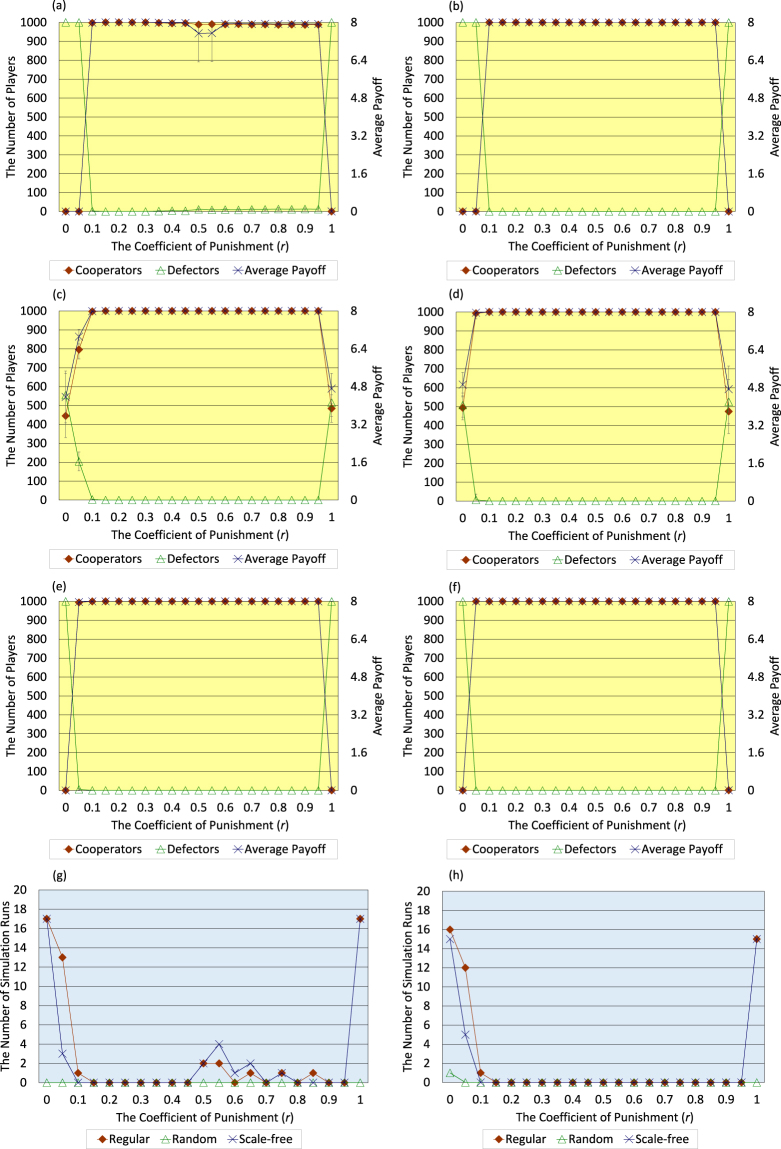
Six panels (a–f) show the dependence of the number of cooperators, the number of defectors, and the average payoff of all players in the 300 generation on the coefficient of punishment (*r*) regarding the cases where the initial topology of connections of <*k*> = 8 is regular (**a**,**b**), random (**c**,**d**), and scale-free (**e**,**f**). Bottom two panels (**g**,**h**) show the dependence of the number of defector-prevailing simulation runs on the coefficient of punishment (*r*) regarding three types of initial topology of connections of <*k*> = 8, i.e. regular, random, and scale-free. Panels of left column (**a,c,e,g**) indicate the results without both activities of breaking and creating connections, and panels of right column (**b,d,f,h**) indicate the results with those activities (error bars: SD, standard deviation). The author decides whether the evolution of cooperation emerges or not on the same basis as described in the figure legend of <*k*> = 4 (Fig. 3).

Secondly, the author describes the results regarding initial regular, random, and scale-free topology of connections of <*k*> = 8. As shown in Fig. 4(a–f), high-level evolution of cooperation and great increase of the average payoff of all players more easily emerge in the case of <*k*> = 8 than in the case of <*k*> = 4 even when both activities of breaking and creating connections are not introduced. Nevertheless, in the case with those activities, the number of cooperators does not slightly decrease when *r* equals 0.5 and 0.55 in the initial regular case (Fig. 4(a,b)). In addition, in the case with those activities, the range of *r* where high-level cooperation evolves and the average payoff of all players greatly increases is wider than such range in the case without those activities in the initial random case (Fig. 4(c,d)). Moreover, as shown in Fig. 4(g,h), in the case with those activities, the number of defector-prevailing simulation runs falls to 0 regarding every initial topology of connections in the range of 0.15 ≤ *r* ≤ 0.95. That range is considerably wider than the range in the case without those activities.

**Figure 5:**
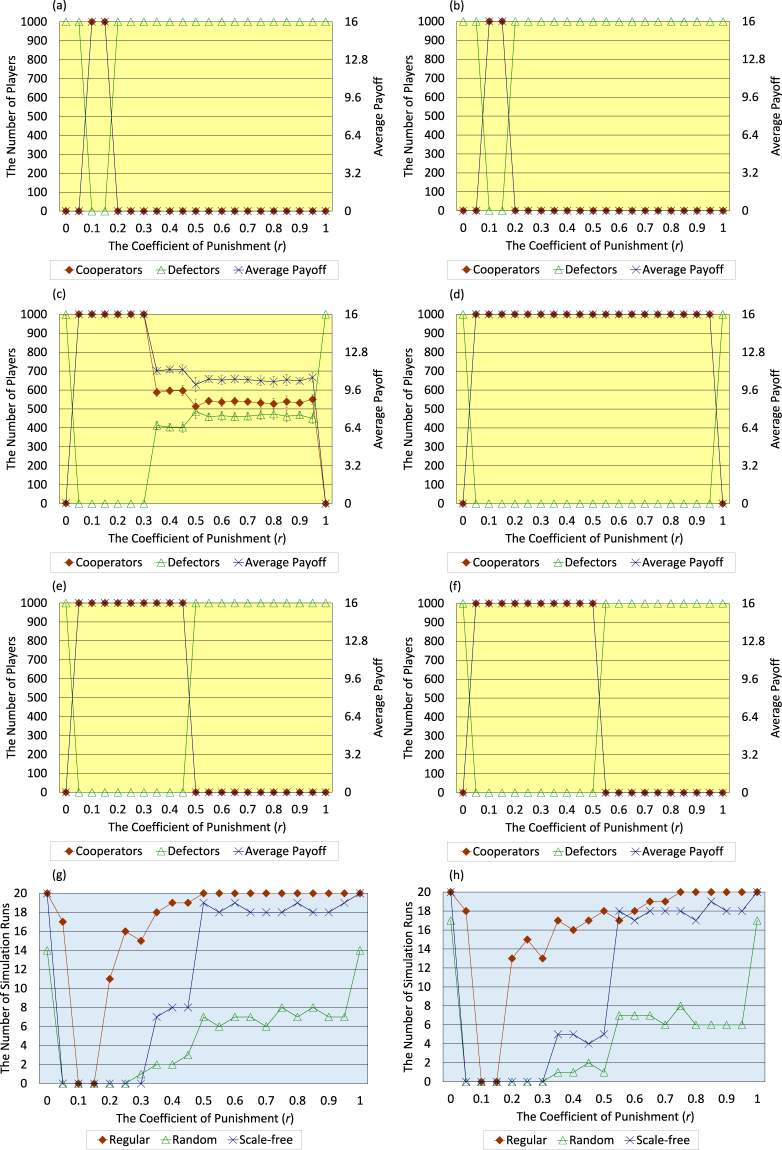
Six panels (a–f) show the dependence of the number of cooperators, the number of defectors, and the average payoff of all players in the 300 generation on the coefficient of punishment (*r*) regarding the cases where the initial topology of connections of <*k*> = 16 is regular (**a**,**b**), random (**c**,**d**), and scale-free (**e**,**f**). Bottom two panels (**g**,**h**) show the dependence of the number of defector-prevailing simulation runs on the coefficient of punishment (*r*) regarding three types of initial topology of connections of <*k*> = 16, i.e. regular, random, and scale-free. Panels of left column (**a**,**c**,**e**,**g**) indicate the results without both activities of breaking and creating connections, and panels of right column (**b**,**d**,**f**,**h**) indicate the results with those activities (error bars: SD, standard deviation). The author decides whether the evolution of cooperation emerges or not on the same basis as described in the figure legend of <*k*> = 4 (Fig. 3).

Finally, the author describes the results regarding initial regular, random, and scale-free topology of connections of <*k*> = 16. As shown in Fig. 5(a–f), high-level evolution of cooperation and great increase of the average payoff of all players are the hardest to emerge in this <*k*> among all cases of <*k*>. Therefore, in the initial regular case (Fig. 5(a,b)), it does not cause any change in the results whether both activities of breaking and creating connections are introduced or not. Nevertheless, the initial random case (Fig. 5(c,d)) exhibits that in the case with those activities, high-level cooperation evolves and the average payoff of all players greatly increases in the range of 0.35 ≤ *r* ≤ 0.95 where such outcome does not emerge in the case without those activities. In addition, in the initial scale-free case (Fig. 5(e,f)), high-level evolution of cooperation and great increase of the average payoff of all players clearly emerge in the case with those activities even when *r* equals 0.5. Moreover, as shown in Fig. 5(g,h), the number of defector-prevailing simulation runs with those activities is smaller than such number without those activities especially regarding the initial random and scale-free cases in the range of *r* ≤ 0.5.

## Discussion

As described in the Results of this study, when introducing both activities of breaking and creating connections of every player to the model of the proposed peer-punishment^1,2^, in the case of <*k*> = 4, 8, the range of *r* where high-level cooperation evolves and the average payoff of all players greatly increases is wider than such range in the case without those activities. In the case of <*k*> = 16, regarding the initial regular case, there is no difference between the results with and without those activities, whereas regarding the initial random and scale-free cases, the number of defector-prevailing simulation runs with those activities is smaller than such number without those activities in the range of *r* ≤ 0.5. The introduction of both activities of breaking and creating connections has a possibility to generate small groups of cooperators that are necessary for the evolution of cooperation because every player keeps connections to cooperators for a long period, whereas he/she frequently breaks connections to defectors. In addition, a defector can keep the connection to a cooperator and exploit him/her only when the peculiar probability at which a defector wants to break connections is greater than that of a cooperator. Moreover, the introduction of the proposed peer-punishment greatly contributes to the stable growth of small cooperative groups. Therefore, cooperation more rapidly evolves in the case with both activities of breaking and creating connections than in the case without those activities.

The author describes the difference between the model of this study and the relevant previous work as follows. Chen *et al*.^33^ propose the model of coevolutionary mechanism in combination with information of cooperative environment (CE) and tolerance threshold of each player. Cooperators can survive and even prevail when they have benign CE around. They show that moderate rationality level can result in the optimal cooperation level. In their model, players unilaterally break the connection to the neighbor player whose lower CE level is out of tolerance threshold, and carry out ordered preferential rewiring. When such rewiring fails, random rewiring is considered. Whereas in this study, players break connections according to the probability that is calculated by their peculiar probability of breaking connections considering their pairing patterns, and create new connections according to the probability of the product of their propensity. Therefore, both activities of breaking and creating connections reflect the preference of each player.

As described in the explanation of the model, in this study, the following events (1) all matches of the prisoner’s dilemma game, (2) all punishing and punished activities, (3) the adoption of new strategy regarding all players, and (4) all activities of breaking and creating connections successively occur, and then topology of connections varies fast. Moreover, there are no additive random payoff disturbances to the payoff of every player. This condition is the reverse of double resonance phenomenon facilitating cooperation presented by Perc^17^, and also not covered by Szolnoki and Perc^23^. This study discovers new knowledge that the combination of probabilistic peer-punishment and coevolutionary mechanism generates small cooperative groups that are necessary for the evolution of cooperation (by coevolutionary mechanism), enlarges such groups (by probabilistic peer-punishment), and as a result, induces high-level evolution of cooperation and great increase of the average payoff of all players in the condition where cooperation is hard to evolve.

Regarding the future work, the author recognizes that the introduction of reward to the proposed model of this study is necessary. Some previous studies^34,35^ present an effect of the coexistence of reward and punishment. For example, Szolnoki and Perc^34^ discuss whether the combined application of reward and punishment is evolutionary advantageous or not, and find rich dynamical behavior that generates intricate phase diagrams where continuous and discontinuous phase transitions successively occur. Chen *et al*.^35^ propose the institutional sanctioning policy that switches the incentive from rewarding to punishing when the frequency of cooperators exceeds a threshold. They find that such policy establishes and recovers full cooperation at lower cost. Therefore, the author plans to investigate an effect of the coexistence of reward, coevolutionary mechanism, and the proposed peer-punishment on the evolution of cooperation. In addition, although the topology of connections becomes roughly random in the end when introducing both activities of breaking and creating connections, the kurtosis of the average degree distribution of lattices regarding the cases of finally all cooperators has proved to be more positive than that regarding the cases of finally all defectors. This phenomenon is observed in the case of <*k*> = 16 regarding the initial random (*r* = 0.75) and scale-free (*r* = 0.5) cases at the moment. The main reason why this phenomenon occurs is that connections to cooperators will last for a long period, whereas those to defectors will frequently rewired. Therefore, it will be necessary to investigate how this phenomenon affects the spatiotemporal dynamics of the evolution of cooperation.
